# A Rare Case of Idiopathic Granulomatous Mastitis in a Nulliparous Woman with Hyperprolactinemia

**DOI:** 10.7759/cureus.4680

**Published:** 2019-05-16

**Authors:** Ankit Agrawal, Sangeetha Pabolu

**Affiliations:** 1 Internal Medicine, Saint Peter's University Hospital - Rutgers Robert Wood Johnson Medical School, New Brunswick, USA; 2 Rheumatology, Saint Peter's University Hospital - Rutgers Robert Wood Johnson Medical School, New Brunswick, USA

**Keywords:** hyperprolactinemia, idiopathic granulomatous mastitis, prolactinoma

## Abstract

Idiopathic granulomatous mastitis (IGM) is a rare, benign, and chronic inflammatory condition of the breast. Women of child-bearing age with a recent history of pregnancy and lactation are usually affected, and clinical picture mimics inflammatory breast cancer or breast abscess. The etiology is not well defined but proposed to be a localized immune reaction to the breast tissue. Here, we report a case of a 41-year-old female who presented with left breast pain and discharge and a clinical diagnosis of breast abscess was made. No improvement with antibiotics was noted and she underwent mammography and diagnostic ultrasound of the affected breast. A biopsy of the lesion was obtained which revealed chronic granulomatous inflammation confirming the diagnosis of IGM. She was also found to have hyperprolactinemia secondary to a prolactinoma following which the patient was started on steroid and bromocriptine.

## Introduction

Idiopathic granulomatous mastitis (IGM), also known as granulomatous lobular mastitis, is an uncommon, benign, and chronic inflammatory condition of the breast. It was first described by Kessler and Wolloch in 1972 [1]. Women of child-bearing age with a recent history of pregnancy and lactation are usually affected [2], and clinical picture mimics inflammatory breast cancer or breast abscess [3]. The etiology is not well defined but proposed to be a localized immune reaction to breast tissue [4]. Gold standard of diagnosis is histopathology. Here, we report a case of a 41-year-old female who presented with breast pain and discharge and subsequently was diagnosed with IGM.

## Case presentation

**Figure 1:**
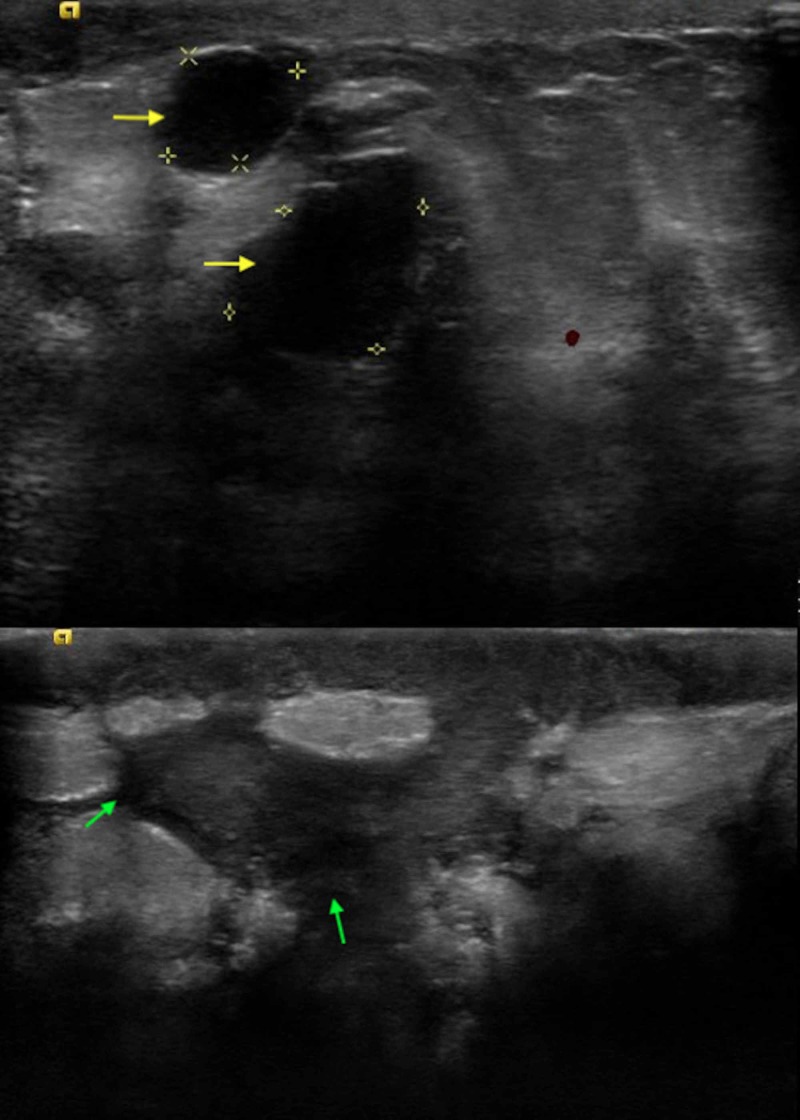
Breast ultrasound depicting multiple complicated cystic lesions (yellow and green arrows).

A 41-year-old female with no past medical history presented to the clinic with left breast pain of two weeks duration. It was associated with a purulent discharge but no fever. She denied any current or previous pregnancies, lactation, or trauma to the breast. On examination, vital signs were within the normal limits. Left breast exam revealed areas of skin excoriation with bleeding and pus on the lower-inner quadrant. A firm lump of 6 cm X 5 cm was palpable underneath the ulcerated area. No abnormal findings were noted on the right breast. Lymph node examination revealed one palpable lymph node in the left axilla. A clinical diagnosis of left breast abscess was made and she was prescribed oral clindamycin for 10 days. She did not notice any improvement in her symptoms and she was evaluated with mammography. It showed inflammatory changes to the solid appearing areas in the regions of skin ulceration. In addition, there were multiple complicated cystic appearing lesions in various locations of the left breast (Figure 1).

**Figure 2:**
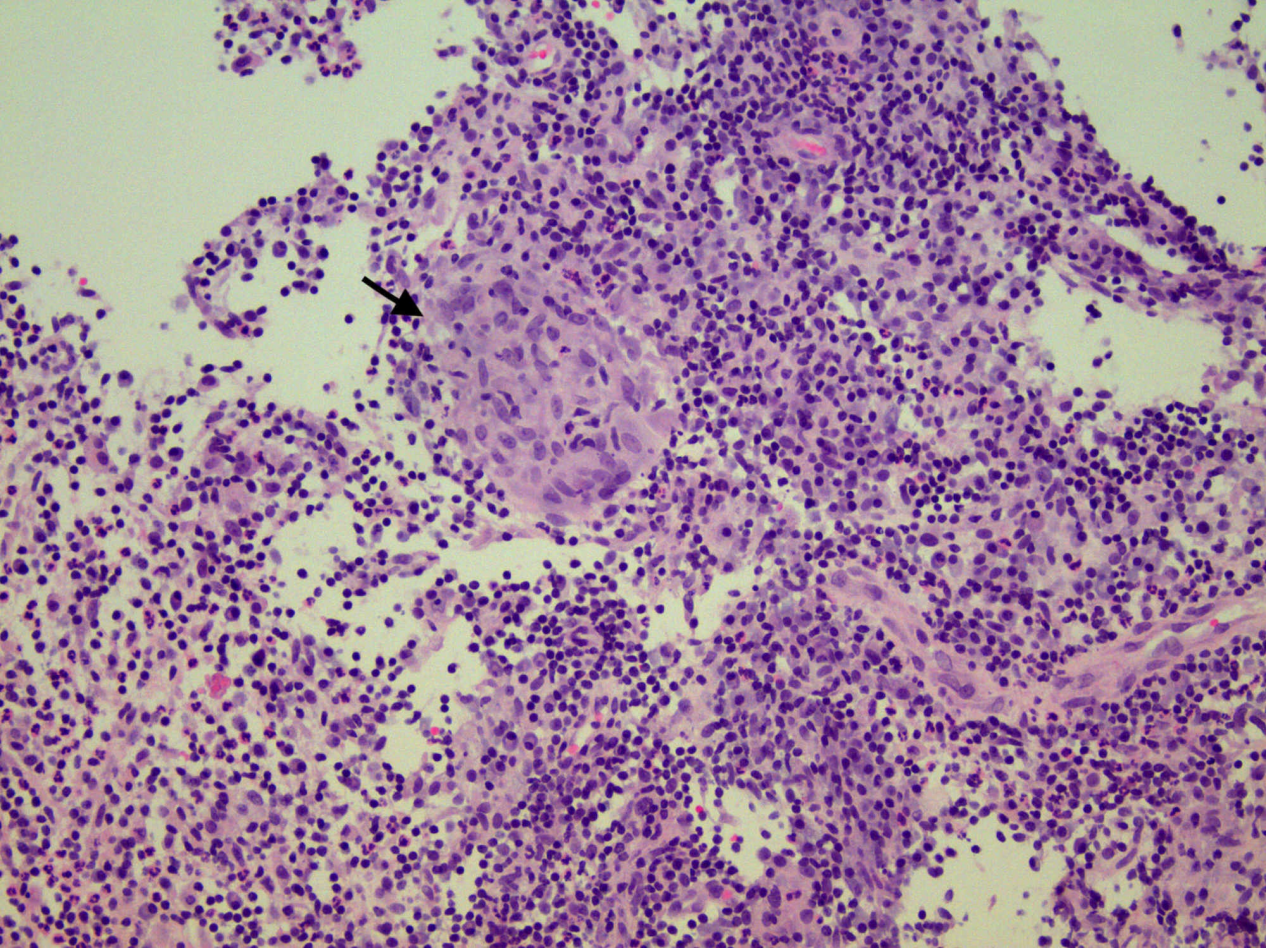
Histopathology showing benign breast tissue with marked acute and chronic inflammation and granulomatous inflammation evident by the presence of noncaseating granuloma (black arrow).

One abnormal left axillary lymph node was noted. The findings were classified under Breast Imaging Reporting and Data System (BI-RADS) assessment category 4 (suspicious abnormality). At this juncture, she was referred to a breast surgeon. Fine needle aspiration (FNA) of the left breast mass and the left axillary lymph node was negative for malignancy. Ultrasound-guided core biopsy of the breast revealed benign breast tissue with acute and chronic inflammation along with noncaseating granulomas and giant cells (Figure 2). 

Special stains were negative for tuberculosis (acid-fast bacilli) and fungal organisms (Histoplasma). Aerobic and anaerobic cultures did not grow any micro-organism including *Corynebacterium kroppenstedtii*.

**Figure 3:**
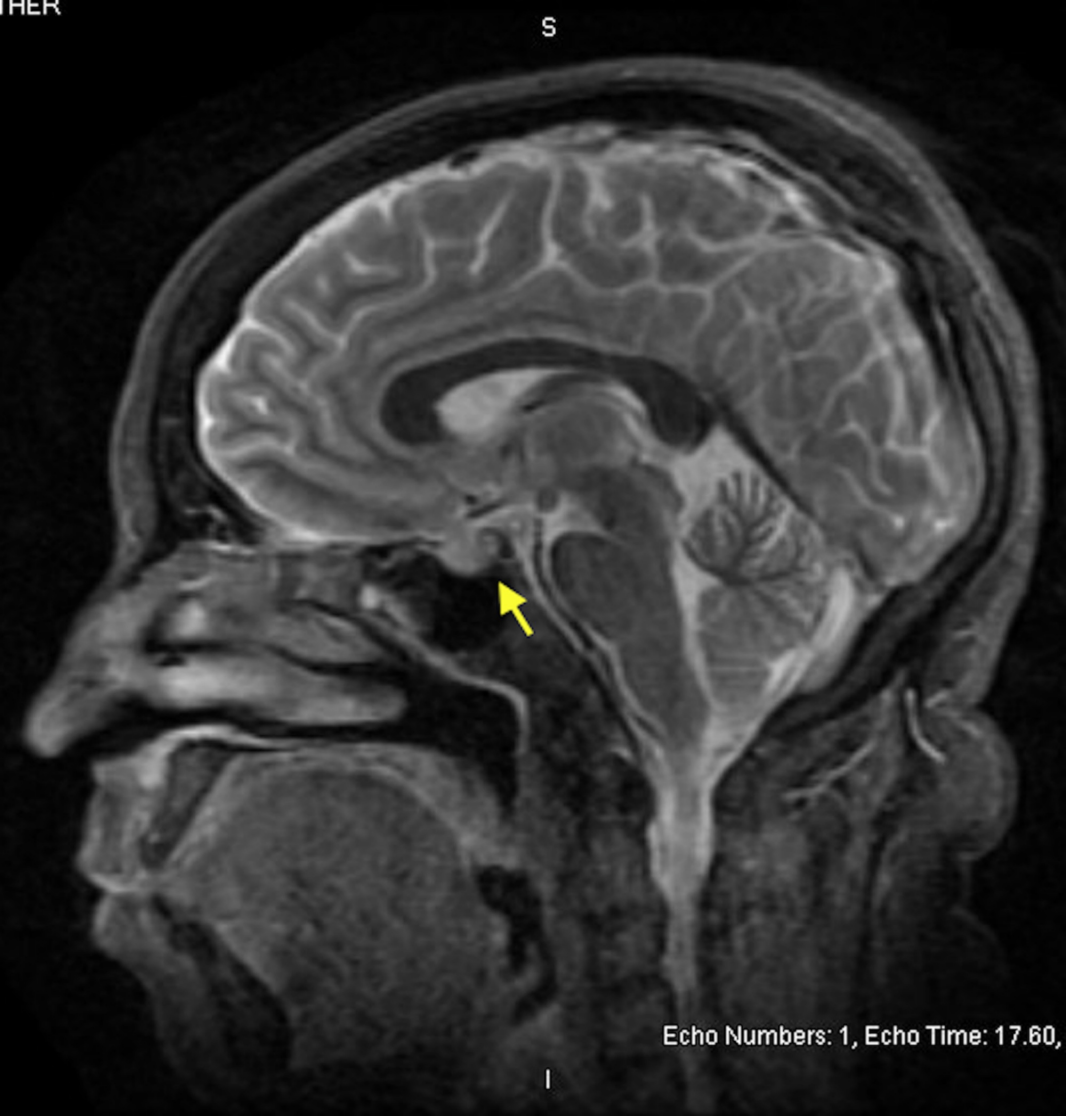
MRI brain in sagittal section showing abnormal enlargement of the pituitary gland (yellow arrow) with extension into the supra-stellar cistern abutting the undersurface of optic chiasm.

The above findings confirmed a diagnosis of granulomatous mastitis and prednisone 0.5 mg/kg/day was initiated. She was referred to rheumatology clinic for further management. Serum anti-neutrophil cytoplasmic autoantibody (ANCA) was negative and serum angiotensin converting enzyme (ACE) level was normal. She was screened for hyperprolactinemia (an associated condition of IGM), and serum prolactin level was found to be elevated at 405.5 ng/mL (normal range for nonpregnant woman: 3-30 ng/mL). A contrast enhanced MRI of brain with detailed imaging of the pituitary gland revealed a pituitary adenoma measuring 11 mm X 10 mm X 12 mm with supra-sellar extension abutting the undersurface of optic chiasm (Figure 3).

The patient responded to oral corticosteroid therapy with significant improvement in pain, discharge, and breast wound. However, due to recurrence of the symptoms with discontinuation of prednisone, she remained on 10 mg prednisone daily at the end of eight months. The plan is to add methotrexate as a steroid sparing agent. She established care with endocrinology for further management of prolactinoma and she was started on bromocriptine 2.5 mg once daily dose.

## Discussion

Idiopathic granulomatous mastitis is a rare and benign chronic inflammatory condition of breast that involves mainly women of child-bearing age. Based on a study by Baslaim et al., histopathological confirmed cases of IGM were 1.8% of cases out of 1,106 women with benign breast condition [5]. The etiology is not well known but has been attributed to chemical reaction associated with oral contraceptive pills, autoimmunity, or localized immune response to extravasation of lobular secretions [3]. An association with local infection with histoplasma and *C. kroppenstedtii* [6] has been proposed as well. Breast trauma, breast feeding, and hyperprolactinemia with galactorrhea are also associated with increased risk of IGM.

Idiopathic granulomatous mastitis mimics inflammatory breast cancer both clinically and radiologically. Patients usually present with a painful progressive breast mass which may cause nipple retraction or inversion and fistulae formation. Peau d’ orange formation is often seen mimicking breast malignancy [5]. It can involve any quadrant sparing the subareolar region. However, a study conducted by Lee et al. demonstrated disease presentation with subareolar involvement in 3 out of 12 case studies [7]. IGM is a diagnosis of exclusion and other conditions that can mimic IGM include breast abscess, breast cancer, foreign body granulomas, Wegener’s granulomatosis, tubercular or fungal mastitis, mammary duct ectasia, and sarcoidosis. Our patient was initially thought to have breast abscess, but it failed to improve with antibiotics.

Most common ultrasonographic finding of IGM is large, irregular hypoechoic mass with multiple tubular extensions, lobulated irregular hypoechoic mass, or distortion of the parenchyma with acoustic shadowing [8]. Our patient had multiple complex cystic lesions along with solid appearing inflammatory regions. The radiological findings have a wide spectrum and it is not limited only to the above-mentioned findings. Dynamic contrast-enhanced MRI has also been suggested to increase specificity of IGM [8]. Although ultrasound and mammography are generally used for the diagnosis, biopsy and histopathological confirmation remains the cornerstone for diagnosis. FNA and core biopsies are usually performed and characteristic findings on histology include noncaseating granulomas, multinucleated Langerhans giant cells, and predominant neutrophilic background with accompanying lymphocytes [3]. Some authors propose that presence of epithelioid histiocytes should raise the suspicion for IGM [9]. Fat necrosis and microabscess formation can also be seen [5].

The management of the IGM is multifold. It can either be monitored with a close follow up in uncomplicated IGM, or can be managed with corticosteroids, immunosuppressive agents, or surgical excision. In a retrospective review study by Lai et al., spontaneous resolution in 50% of IGM cases was demonstrated [10]. There is no role of antibiotics in management. High dose corticosteroids can be administered for the treatment of IGM and steroid sparing agents like methotrexate can be introduced to avoid the long-term adverse effects of steroids and to facilitate their weaning.

## Conclusions

Breast lumps or abscesses are commonly encountered in clinical practice. IGM, although rare, is an important entity which should not be missed in evaluating an inflammatory condition of the breast especially in women of child-bearing age (pregnant or breast feeding). The chronicity and lack of response to antibiotics should raise the suspicion for IGM. While imaging studies may assist in diagnosis, biopsy remains the cornerstone in establishing the diagnosis. Due to its association with hyperprolactinemia, screening prolactin is recommended, and if elevated, can prompt timely diagnosis and management of pituitary adenoma as reported in our patient. Corticosteroids and immunosuppressive agents such as methotrexate are very effective in management of IGM and in majority of cases avoid the need for surgery and potential complications such as fistula formation and disfigurement after surgery. Compared to prior published literature, unique attributes in our case are diagnosis of IGM in nulliparous woman with hyperprolactinemia without the usual association of *C. kroppenstedtii* infection.
